# Impact of cervical full-endoscopic spine surgery on intracranial pressure in swine

**DOI:** 10.1016/j.bas.2026.107442

**Published:** 2026-09-09

**Authors:** Marcelo Campos Moraes Amato, Vinicius Marques Carneiro, Denylson Sanches Fernandes, André Cleriston José dos Santos, Ricardo Santos de Oliveira

**Affiliations:** a Division of Neurosurgery, Department of Surgery and Anatomy, Ribeirão Preto Medical School, University of São Paulo, Ribeirão Preto, Brazil; b Division of Neuromuscular Disorders, Department of Neurology, Ribeirão Preto Medical School, University of São Paulo, Ribeirão Preto, Brazil

**Keywords:** Full-endoscopic spine surgery, Saline irrigation, Intracranial pressure, Anesthesia depth, Cervical spine, Swine model

## Abstract

**Introduction:**

Cervical full-endoscopic spine surgery (FESS) has grown in clinical use, but the effects of irrigation on intracranial pressure (ICP) remain poorly understood and may pose neurological risks.

**Research question:**

Whether cervical FESS induces relevant ICP increases compared with prior lumbar data, under different irrigation parameters, drainage occlusion states, and anesthesia depths.

**Material and methods:**

In a controlled swine model, four female *Sus scrofa domesticus* (50–75 kg) underwent cervical FESS with a uniportal, dual-channel suction-irrigation endoscope under total intravenous anesthesia. ICP was monitored invasively via intraparenchymal catheter. Irrigation pump settings were tested in graded configurations with drainage outflow open, partially occluded, or totally occluded. Anesthesia depth was modulated to assess its influence on ICP dynamics.

**Results:**

Baseline ICP averaged 11.1 mmHg. Cervical FESS produced marked ICP increases, particularly during partial and total drainage obstruction, with peaks up to 102 mmHg. Under open drainage, ICP ranged from 7.9 to 29.1 mmHg, occasionally exceeding the 20 mmHg safety threshold but remaining lower than during occlusion. With anesthesia-depth modulation, mild sedation yielded occlusion peaks up to 62.5 mmHg, whereas deeper anesthesia was associated with open-drainage ICP of 9.5–19.7 mmHg and occlusion peaks of 23–36.6 mmHg.

**Discussion and conclusion:**

Cervical FESS was associated with earlier and more pronounced ICP elevations than lumbar procedures. Saline outflow occlusion is the primary trigger of critical pressure surges, while pump parameters modulate magnitude. Deeper anesthesia was associated with attenuated ICP responses. Maintaining drainage patency, using the lowest effective pump settings, and appropriate anesthetic management may reduce the risk of critical intracranial hypertension.

## Highlights

• Cervical full-endoscopic spine surgery increases intracranial pressure.
• Saline outflow obstruction triggers critical intracranial pressure elevations.
• Patent drainage limits ICP rise despite high pump settings.
• Deeper anesthesia was associated with attenuated intracranial pressure surges.
• Experimental swine model clarifies irrigation safety limits.

## Abbreviations

FESS: Full-Endoscopic Spine Surgery
ICP: Intracranial Pressure
CSI: Continuous Saline Irrigation
TIVA: Total Intravenous Anesthesia
OpDC: Opened Drainage Channels
PODC: Partial Occlusion of Drainage Channels
TODC: Total Occlusion of Drainage Channels
MAP: Mean Arterial Pressure
CRS: Clinically Representative Scenario

## 1 Introduction

Full-endoscopic spine surgery (FESS) has expanded substantially over the past decade and is now widely adopted for the treatment of degenerative spinal disorders (Hofstetter et al., 2020a; Muthu et al., 2021; Birkenmaier et al., 2013; Ruetten et al., 2018). Its application in cervical disc herniations and foraminal stenosis has transitioned from experimental surgery to routine clinical practice in numerous centers worldwide (Ruetten et al., 2008, 2018; Lee et al., 2018a, 2018b; Birjandian et al., 2017; Kim et al., 2017a; Gibson et al., 2021; Komp et al., 2018). The reported advantages of FESS include reduced soft-tissue disruption, minimal intraoperative bleeding, accelerated postoperative recovery and low rates of surgical site infection (Choi et al., 2016; Marković et al., 2020). Most notably, the magnified endoscopic view provides superior visualization of neural and bony structures, which may translate into improved surgical precision and enhanced procedural safety. These characteristics have made posterior cervical foraminotomy particularly well suited to a full-endoscopic approach, with growing evidence demonstrating favorable clinical outcomes (Komp et al., 2018; Bucknall and Gibson, 2018; Wu et al., 2018; Carr et al., 2020).

However, complications do occur and may be particularly harmful in cervical FESS due to the proximity of critical neurovascular structures (Vargas et al., 2023a). Other adverse effects are well reported and may be related to elevated intracranial pressure (ICP) due to continuous saline infusion (CSI) into the epidural space (Amato et al., 2023): headache, neck pain, hypoacusis, and paresthesias (Joh et al., 2009; Flaviano and Bellini, 2018), and more severe, although rare, such as visual loss, retinal hemorrhages (Gill and Heavner, 2005; Moschos et al., 2008), and epileptic seizures (Choi et al., 2011; Lin et al., 2020). Continuous saline irrigation, particularly with pumps allowing control of pressure and flow, is essential for bleeding control and surgical visualization (Amato, 2023; Hofstetter et al., 2020b) and should not be discouraged. Nonetheless, a deeper understanding of the relationship between CSI and ICP elevation may enable surgeons to optimize irrigation strategies and improve procedural safety. Our previous study demonstrated that ICP elevation during lumbar FESS is most pronounced when drainage channels are occluded — a maneuver commonly used to control bleeding and improve the surgeon's view — and that this effect is exacerbated at higher pump pressures (Amato et al., 2023). Uncertainties remain regarding the specific impact of cervical FESS on ICP, particularly given its closer anatomical relationship to the intracranial compartment. An additional observation from our previous work was that increases in ICP occurred not only with complete outflow occlusion but also with partial occlusion during the intraoperative use of bulky instruments, such as Kerrison rongeurs or endoscopic burrs, during routine decompression maneuvers. Furthermore, the observation that similar intraoperative events produced different ICP responses across animals in our previous porcine experiments raised the possibility that anesthetic depth might modulate ICP, potentially through its effects on cerebral physiology.

Based on these considerations, the present study aimed to characterize the invasive ICP profile during cervical FESS in swine, compare these findings with our previous lumbar spine experiments, evaluate the influence of different irrigation pump parameters on ICP across distinct outflow conditions (open drainage, partial occlusion, and total occlusion), and determine whether anesthetic depth modulates these ICP responses.

## 2 Methods

This experimental study in swine was approved by the Institutional Animal Care and Use Committee (protocol n. 1058/2022) and conducted in accordance with institutional guidelines and Brazilian regulations for animal experimentation. All procedures complied with the ARRIVE guidelines and internationally accepted standards for the care and use of laboratory animals.

### 2.1 Equipment

**Fig. 1:**
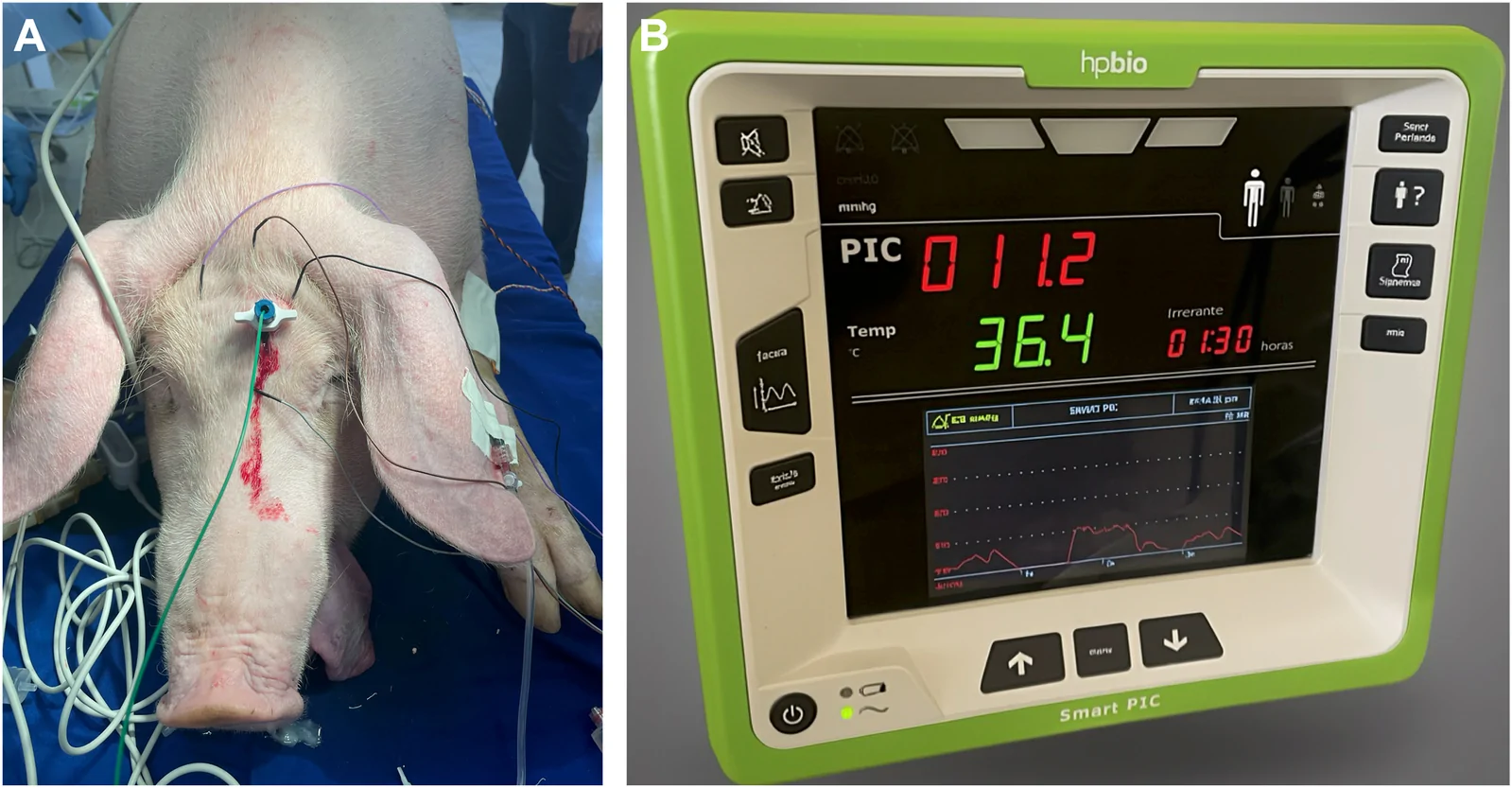
A) Swine 1 anesthetized and positioned prone, with an invasive ICP catheter inserted through a frontal burr hole in the skull. B) ICP monitor displaying a baseline value of 11.2 mmHg immediately after ICP catheter placement.

**Fig. 2:**
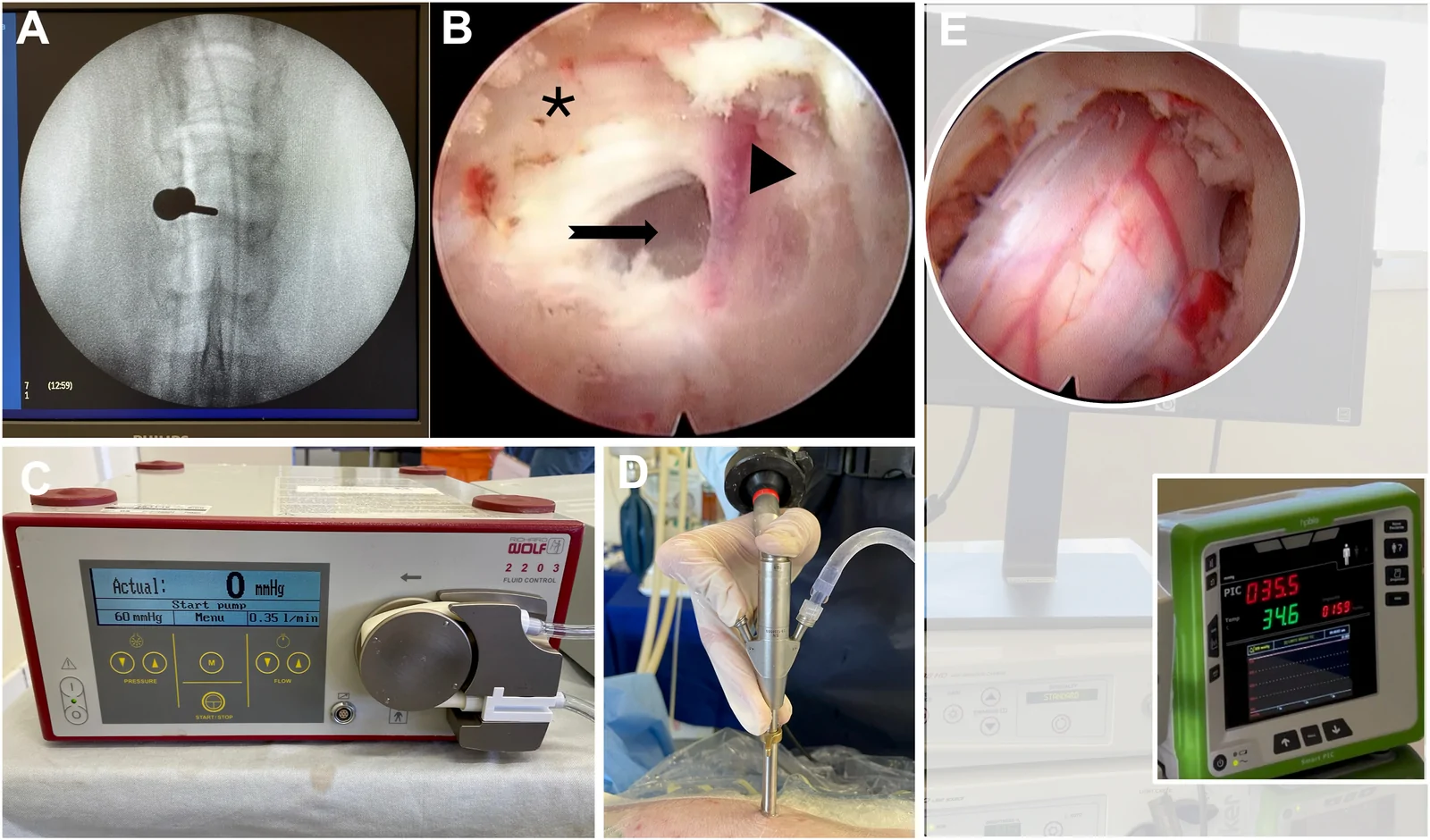
A) AP fluoroscopic image showing cannula insertion directed toward the C4-C5 interlaminar window. B) Endoscopic view showing partial cranial laminectomy and initial flavectomy (*: ligamentum flavum; arrowhead: cranial lamina; arrow: spinal canal) C) Irrigation pump set to configuration A: 60 mmHg and 350 mL/min. D) Uniportal transforaminal endoscope used in the experiments, showing the two lateral 1.5-mm suction-irrigation channels and manual occlusion of the outflow channels. E) Monitor displaying the endoscopic view of the spinal cord and nerve root after cervical laminectomy and flavectomy, along with the ICP monitor showing a value of 35.5 mmHg immediately following total occlusion of the drainage channels.

Equipment included an ICP monitor and intraparenchymal catheters (HPBio®); a Fluid Control 2203 automatic irrigation pump (Richard Wolf®) (Fig. 1A); a Stryker® video tower with monitor, light source, shaver, camera control and recorder; and a C-arm (Ziehm® solo). A uniportal full-endoscopic system was used (Doppkon GmbH & Co. KG, Spaichingen, Germany), equipped with a transforaminal endoscope (A-100-611; outer diameter 7.0 mm, working length 181 mm, 4.3-mm working channel, 30° viewing angle) featuring two independent 1.5-mm internal-diameter lateral suction-irrigation channels. During irrigation, one lateral channel was connected to the pump, while saline outflow occurred through the contralateral lateral channel and through the working channel when not occupied by an instrument (Fig. 2D). The transforaminal endoscope had the same outer diameter and working-channel diameter as the interlaminar endoscope used in our previous study, but had a longer working length, which was necessary to reach the spinal canal through the thicker cervical soft tissues of swine.

Although the irrigation pump had a valid recent calibration, fine calibration was performed before the experiments using the same instruments and devices employed during the procedures to verify that the volume of saline delivered over 1 min corresponded to the programmed flow-rate setting.

### 2.2 Experimental model and anesthesia

Four female *Sus scrofa domesticus*, 4 months old and weighing 50–75 kg, were included in the study and managed by the medical school's experimental laboratory staff. Only female animals were used, and sex-related differences were not specifically analyzed. The animals were continuously monitored and underwent total intravenous anesthesia (TIVA) and tracheostomy, with ventilatory support provided and adjusted as required by two experienced veterinarians. The anesthetic protocol was designed to replicate the regimen commonly used by the authors in human full-endoscopic spine surgery (FESS), consisting of total intravenous anesthesia without neuromuscular blockade. Mild sedation was achieved with Propofol (2000 mcg/kg/h) and Fentanyl (5 mcg/kg/h), while deeper sedation was induced by increasing infusion rates to 6000 mcg/kg/h and 10 mcg/kg/h, respectively. Anesthetic depth was assessed based on apnea induction and blood pressure reduction. Additional details regarding the anesthetic protocol are provided in Table 1. It is important to note that Bispectral Index monitoring was employed to assess anesthesia depth; however, the readings proved inconsistent and could not be considered reliable. Consequently, these values are not reported.

**Table 1:** Anesthetic strategy. Summary of animal weight, anesthetic agents administered, duration of continuous saline irrigation (CSI), peak intracranial pressure (ICP) recorded during partial (PODC) and total (TODC) outflow occlusion, Clinically Representative Scenario (CRS) values—defined as the highest ICP measured in Configuration A under partial and total occlusion—and relevant individual observations.

Pig	Weight (kg)	Anesthesia	CSI Duration	Highest ICP with Partial and Total occlusion of outflow (mmHg)	Observations
**1**	74	Induction with Ketamine 1g and Xylazine 200 mg. Propofol at 4000mcg/kg/h, Fentanyl at 5mcg/kg/h, Tramadol 1amp: MILD SEDATION	1h35′ From 10:05 to 11:40am	PODC: 60.7(Config C at 10:47am) TODC: 102 (Config D at 11:00am) CRS (P: 33.1 and T: 40.4)	This pig exhibited high physiological resilience, and despite attempts to deepen anesthesia, with high doses of anesthetic drugs, it did not induce apnea or reduce blood pressure.
**2**	65	Induction with Ketamine 1g and Xylazine 200 mg. Propofol at 6000mcg/kg/h. Fentanyl at 10mcg/kg/h: DEEP SEDATION	1h07′ From 1:57 to 3:04pm	PODC: 25.2 (Config A at 2:11pm) TODC: 26.3 (Config A at 2:17pm) CRS (P: 25.2 and T: 26.3)	In contrast to the first animal, the anesthetic regimen produced deep and stable sedation, with lower ICP variability across pump settings and drainage occlusion.
**3**	52	Induction with Ketamine 500 mg and Xylazine 80 mg. Followed by Propofol at 2000mcg/kg/h, Fentanyl at 5mcg/kg/h: MILD SEDATION	1h14′ From 9:16 to 10:30am	PODC: 60.1 (Config B at 09:43am) TODC: 62.5 (Config B at 09:46am) CRS: (P: 33 and T:44.2)	Anesthesia induction was performed with lower intramuscular doses of ketamine and xylazine, which are required to allow venous access. Although this strategy was challenging, it enabled better control of intravenous anesthesia, allowing us to achieve mild sedation initially and then deepen anesthesia by adjusting propofol and fentanyl doses.
		Then for further sedation: Propofol at 6000mcg/kg/h and Fentanyl at 10mcg/kg/h: DEEP SEDATION	33′ From 10:30 to 11:03am	PODC: 21.8 (Config B at 10:35am) TODC: 36.3 (Config D at 10:54am) CRS (T: 23.7)	
			Total: 1h47′		
**4**	50	Induction with Ketamine 500 mg and Xylazine 80 mg. Followed by Propofol at 2000mcg/kg/h, Fentanyl at 5mcg/kg/h: MILD SEDATION	1h01′ From 12:34 to 1:35pm	PODC: 28.8 (Config B at 12:53pm) TODC: 54 (Config C at 1:03pm) CRS: (P: 18.4 and T: 31.2)	The same strategy used for pig no. 3 allowed testing of both mild and deep sedation. However, maintaining deep sedation throughout the procedure proved challenging, as the animal repeatedly exhibited elevated blood pressure and spontaneous respiratory effort during the deep-sedation tests. Results according to the animal's physiological state.
		Then for further sedation: Propofol at 6000mcg/kg/h and Fentanyl at 10mcg/kg/h: DEEP SEDATION	55′ From 1:35 to 2:30pm	PODC: 30.9 (Config D at 2:08pm) TODC: 36.6 (Config D at 2:13pm) CRS (P: 23 and T: 23.4)	
			Total: 1h56′		

### 2.3 Surgical technique

The animals were positioned prone on a radiolucent surgical table. A frontal scalp incision was performed, followed by a frontal burr hole to allow placement of an intraparenchymal catheter for invasive ICP monitoring (Fig. 1A). The catheter was connected to the ICP monitor (HPBio®), and baseline ICP values were recorded (Fig. 1B). An incision was made, and a dilator was inserted toward the C4–C5 or C5–C6 interlaminar space under fluoroscopic guidance (Ziehm® Solo C-arm) (Fig. 2A). The working cannula and irrigated endoscope were then introduced. At this stage, the irrigation pump had already been configured with the predefined initial parameters. An interlaminar approach was performed, including partial laminectomy, partial facetectomy, and flavectomy sufficient to expose the dural sac and nerve root, allowing safe introduction and free manipulation of instruments within the spinal canal**.** Irrigation time was recorded from the initiation of endoscopic irrigation. Spinal canal opening was defined as the first opening of the ligamentum flavum (Fig. 2B).

### 2.4 Tests and experimental conditions

Continuous saline irrigation (CSI) using an automatic irrigation pump was maintained throughout the procedures. Irrigation pressure parameters were progressively increased according to four predefined configurations:

• **Configuration A:** 60 mmHg/350 mL/min
• **Configuration B:** 90 mmHg/350 mL/min
• **Configuration C:** 120 mmHg/700 mL/min
• **Configuration D:** 150 mmHg/700 mL/min

Intracranial pressure (ICP) and systemic hemodynamic parameters were continuously recorded during each configuration.

Each pump setting was tested under three drainage-channel conditions:

• Open drainage channels (OpDC): measurements obtained while no instrument occupied the working channel, allowing saline outflow through both the free lateral suction-irrigation channel and the working channel.
• Partial occlusion of drainage channels (PODC): measurements obtained during routine use of large-diameter instruments, such as Kerrison punches or endoscopic burrs. Because these instruments occupied the working channel almost completely, saline outflow occurred predominantly through the second lateral suction-irrigation channel.
• Total occlusion of drainage channels (TODC): complete outflow obstruction produced manually by occluding the free lateral suction-irrigation channel and the working channel simultaneously, as illustrated in Fig. 2D.

The planned protocol included approximately 2 min of open drainage, up to 5 min of occlusion, and approximately 3 min of recovery. In practice, occlusion was generally discontinued once a stable ICP plateau was reached, usually within 2–3 min, and subsequent testing began only after ICP had returned to a new stable plateau. PODC duration was less uniform because measurements were obtained during actual surgical maneuvers; these periods were longer during the initial configurations, while decompression was still being performed, and generally lasted 2–3 min during the later configurations.

**Fig. 3:**
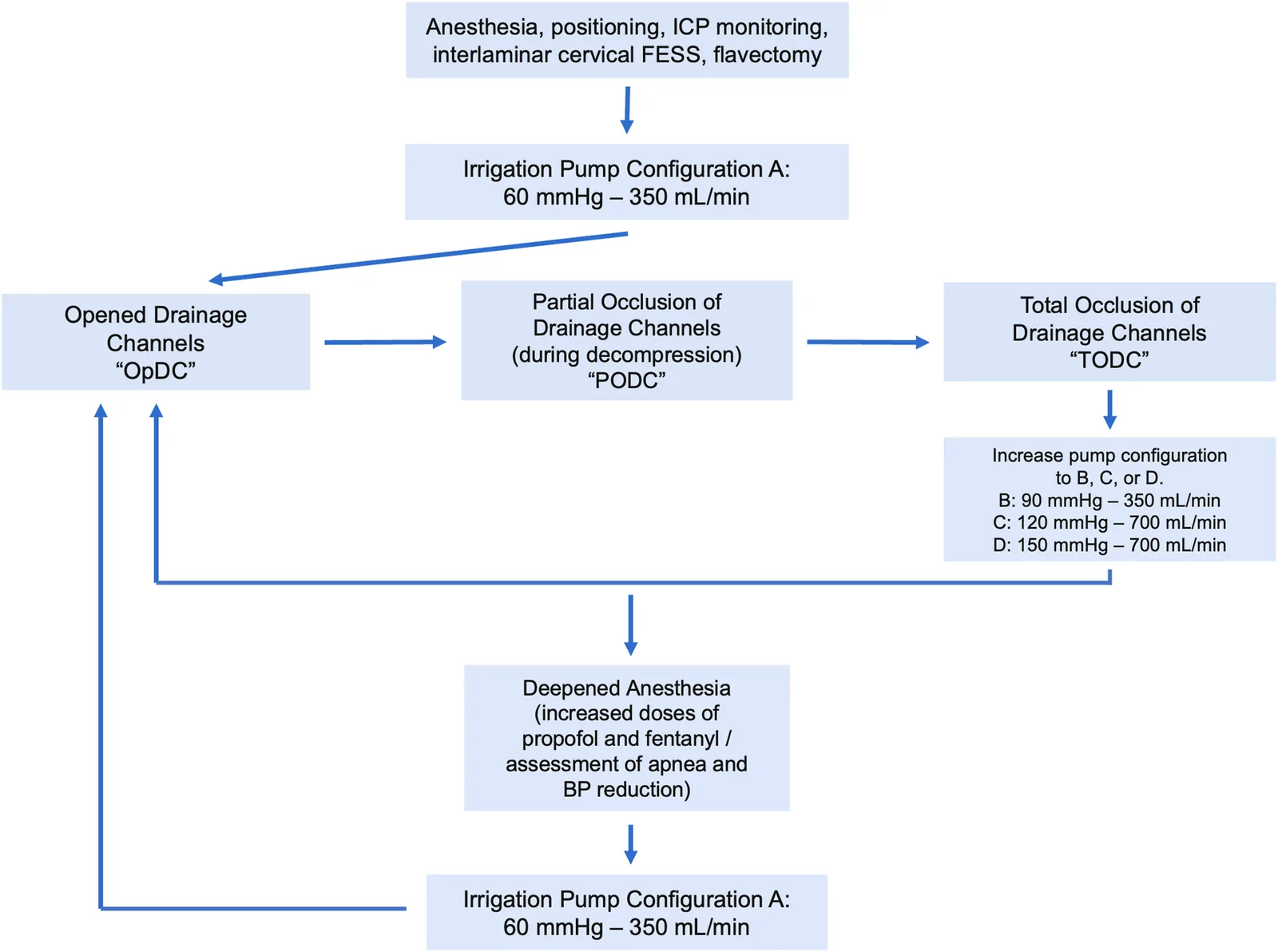
Flowchart of the experimental steps.

Anesthetic depth was tested at the predefined mild and deep levels described above. ICP and arterial blood pressure were continuously recorded throughout all experimental phases. The overall experimental workflow is illustrated in Fig. 3. Safety was defined as the absence of sustained ICP elevation above 20 mmHg.

## 3 Results

**Fig. 4:**
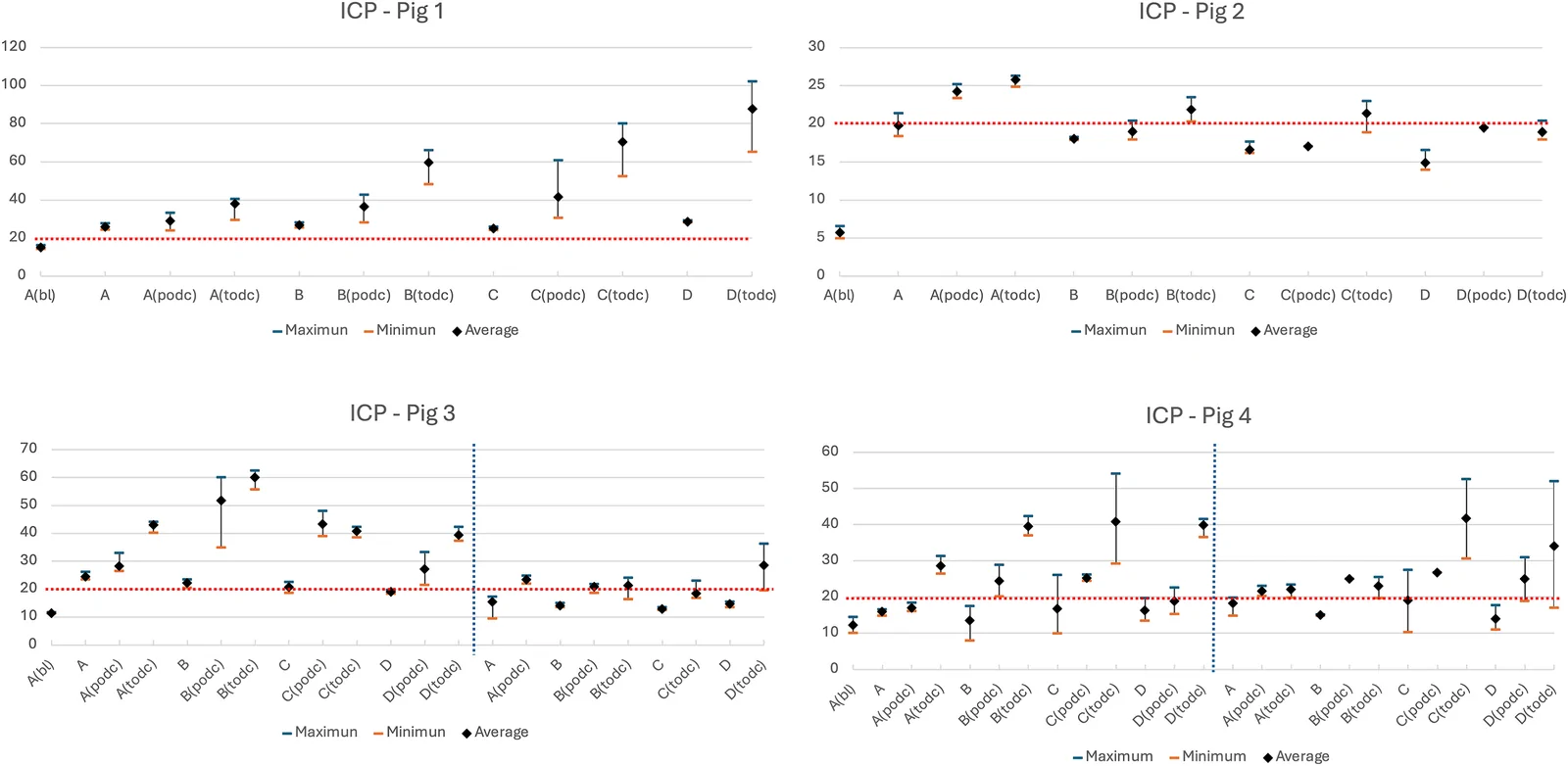
Intracranial pressure (ICP) profiles for each pig. Minimum, maximum, and average ICP values are presented according to irrigation pump configuration and drainage channel status: open, PODC (partial occlusion of drainage channels), and TODC (total occlusion of drainage channels). Pump configurations were: A, 60 mmHg and 350 mL/min; B, 90 mmHg and 350 mL/min; C, 120 mmHg and 700 mL/min; and D, 150 mmHg and 700 mL/min. BL indicates baseline. The red dotted line at 20 mmHg indicates the upper safety threshold. Where applicable, the blue dotted line separates measurements obtained during the protocol-defined mild and deep anesthesia phases.

**Fig. 5:**
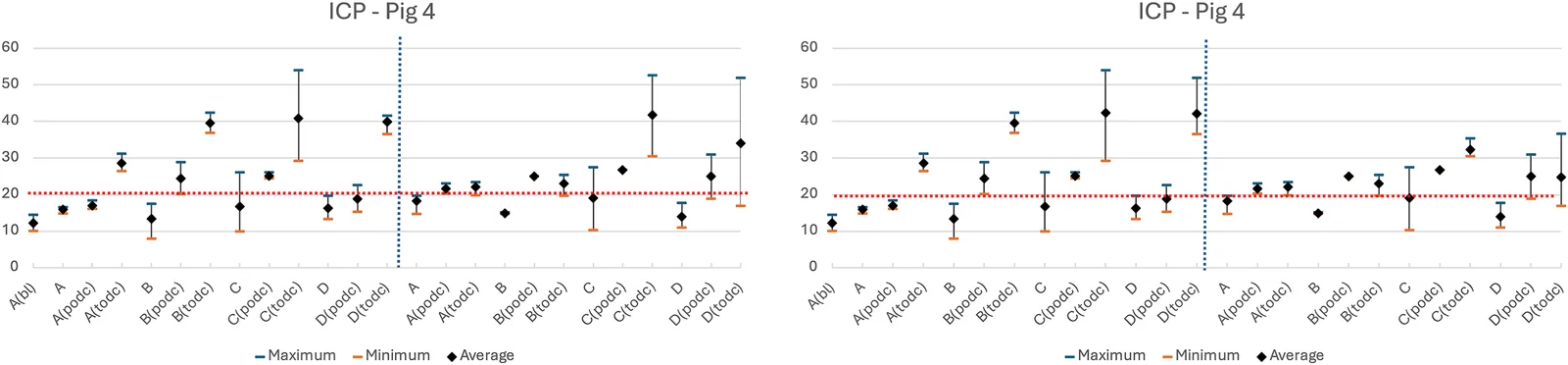
ICP values according to anesthetic depth in Pig 4. **Left**: Data displayed according to the protocol-defined mild and deep anesthesia phases. **Right**: Data reclassified according to the animal's physiological state, based on respiratory drive and blood pressure, to account for transient variations in anesthetic depth. In each panel, the blue dotted line separates measurements classified as mild and deep sedation according to the respective criterion. The red dotted line at 20 mmHg indicates the upper safety threshold. BL indicates baseline.

**Fig. 6:**
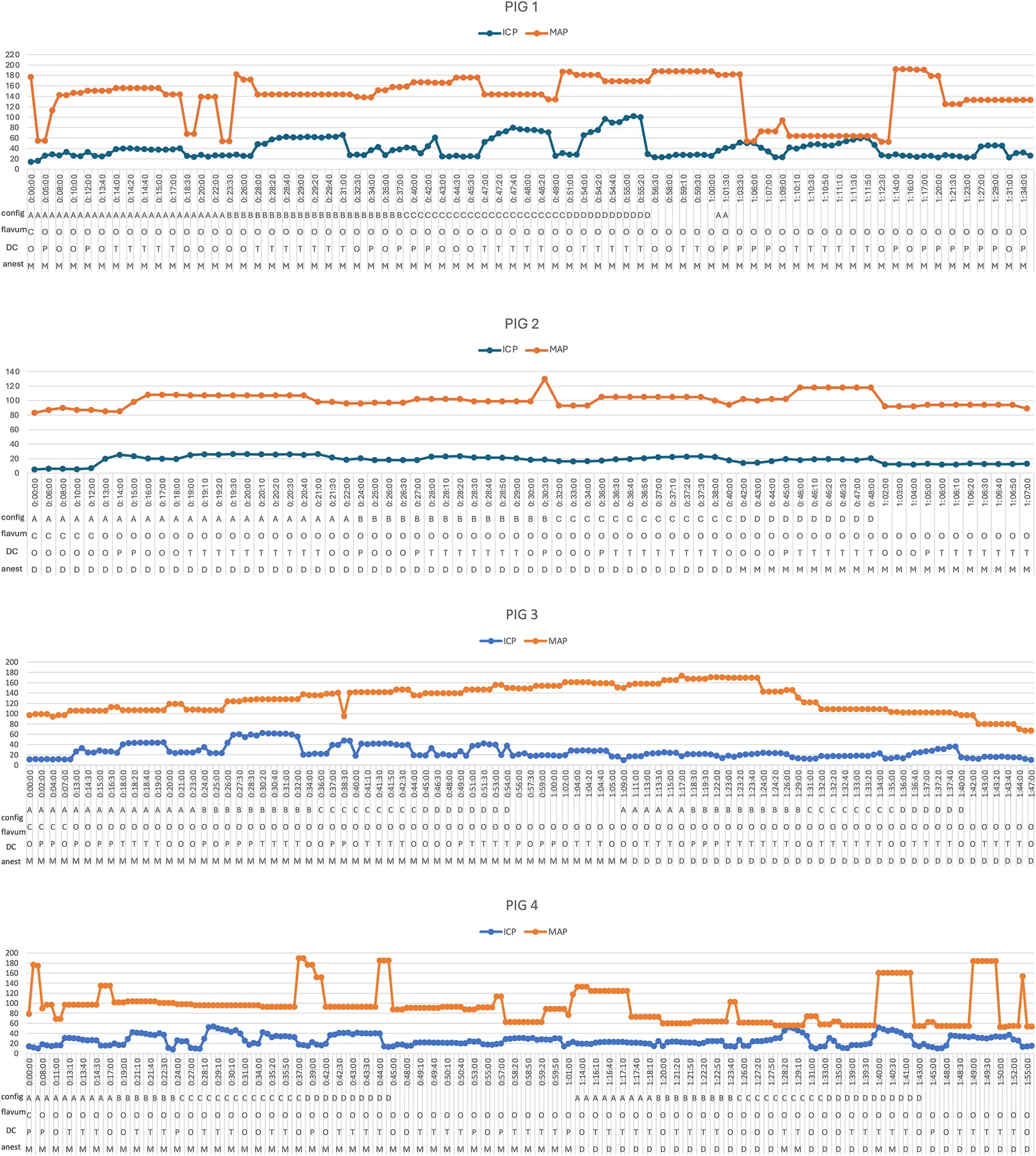
ICP (blue) and MAP (orange) profiles for each pig over time. The graphs display temporal evolution according to pump configuration (A to D), vertebral canal status (flavum closed [C] or opened [O]), drainage channel condition (open [O], partial occlusion [P], or total occlusion [T]), and depth of anesthesia (mild [M] or deep [D]).

All four animals maintained stable clinical status during the procedures; however, as in human surgery, adjustments to anesthetic dosing or minor modifications to the testing protocol were occasionally required in response to marked hemodynamic alterations or signs of discomfort (Table 1). Baseline ICP, recorded immediately after placement of the parenchymal intracranial catheter, averaged 11.1 mmHg. The results described below are further illustrated in Fig. 4, Fig. 5, Fig. 6.

In pig 1, intracranial pressure (ICP) rose above 20 mmHg immediately after opening the ligamentum flavum and remained elevated across all irrigation pump settings with open drainage (OpDC). Partial (PODC) and total drainage occlusion (TODC) produced critical ICP elevations, with peak values reaching 40.4 mmHg in Configuration A and escalating to 66, 80.1, and up to 102 mmHg in configurations B, C, and D, respectively. Notably, this animal exhibited marked physiological resilience; it was the heaviest of the group (74 kg), and despite attempts to deepen anesthesia with high doses of anesthetic agents, apnea could not be induced, and blood pressure did not decrease.

In pig 2, average ICP remained below 20 mmHg with open drainage across all pump settings, whereas partial and total occlusion resulted in only mild ICP elevations (>20 but <27 mmHg), a pattern that contrasted sharply with pig 1. Also contrasting with pig 1, the anesthetic regimen produced deep and stable sedation, with lower ICP variability across pump settings and occlusion maneuvers. After 40 min, anesthetic administration was discontinued in an attempt to achieve mild sedation; however, although the animal moved, neither MAP nor ICP increased.

In pigs 3 and 4, it was possible to test different depths of anesthesia. Under mild sedation, pig 3 demonstrated OpDC ICP values of 18.3–26.2 mmHg and PODC/TODC peaks of 44.2, 62.5, 48, and 42.3 mmHg (Configurations A–D, respectively), with ICP during occlusion reaching 62.5 mmHg in Configuration B; deeper sedation lowered OpDC ICP to 9.5–17.3 mmHg and reduced PODC/TODC peaks to 24.9, 24.1, 23, and 36.3 mmHg. Deeper sedation was associated with lower ICP when the outflow was open and with reduced variability during occlusion tests.

Similarly, pig 4 under mild sedation demonstrated OpDC ICPs of 7.9–26 mmHg and PODC/TODC peaks of 31.2, 42.3, 54, and 51.9 mmHg, whereas deeper sedation resulted in OpDC ICPs of 10.9–19.7 mmHg and PODC/TODC peaks of 23.4, 25.4, 35.4, and 36.6 mmHg. During deep anesthesia, apnea required manual ventilation by the veterinary anesthesiologist. Increased ventilation was followed by an immediate reduction in ICP, observed after an ICP peak during deep sedation. In pig 4, due to intermittent discontinuation of anesthetic agents to adjust depth, transient episodes of lighter anesthesia occurred within the planned deep-sedation phases. Accordingly, values were stratified according to the animal's physiological response (respiratory drive and blood pressure), defining mild and deep anesthesia based on observed clinical parameters rather than predefined protocol timing. Fig. 5 presents both the protocol-defined analysis and the physiology-based reclassification, showing lower ICP peaks during occlusion phases classified as deep anesthesia.

The mean time from initiation of endoscopic irrigation to the first opening of the ligamentum flavum, i.e., spinal canal opening, was 9.75 min. Total procedure duration ranged from 67 to 116 min (mean, 96.25 min) and was longer in pigs 3 and 4 (107 and 116 min, respectively), as these animals allowed testing of different depths of anesthesia.

With open drainage, the observed pump-system pressure remained between the preset pressure limit and approximately 5 mmHg below that limit, indicating that the delivered flow was at or slightly below the programmed value. During occlusion tests, the observed system pressure reached the preset limit, meaning infusion was minimal to maintain that pressure.

Fig. 4 illustrates the minimum, maximum, and average intracranial pressure (ICP) values according to irrigation pump configuration and drainage channel status, whereas Fig. 6 depicts the temporal evolution of ICP and mean arterial pressure (MAP) profiles for each pig over time.

## 4 Discussion

### 4.1 Irrigation, outflow and ICP

Continuous saline irrigation (CSI) is a fundamental component of full-endoscopic spine surgery, providing a clear operative field, effective hemostasis, and continuous visualization of neural and bony structures. As endoscopic techniques have evolved, increasingly complex procedures have become possible, often requiring longer periods of continuous irrigation (Hofstetter et al., 2020a; Birkenmaier et al., 2013; Amato, 2023; Teli et al., 2010; Arts et al., 2009). Current indications include direct neural decompression, high-speed drilling, treatment of stenosis and tumors, and selected stabilization procedures, all of which may substantially increase irrigation-dependent operative time (Lee et al., 2018a; Birjandian et al., 2017; Ruetten et al., 2006, 2008; Gibson et al., 2021; Komp et al., 2018; Cheng et al., 2014; Kim et al., 2017b; Kotheeranurak et al., 2022; Kravtsov et al., 2022; Telfeian et al., 2015; Tsai et al., 2018). Together with increasing reports of neurological complications potentially related to irrigation, this evolution has raised concerns regarding the hydrodynamic safety of FESS (Joh et al., 2009; Flaviano and Bellini, 2018; Gill and Heavner, 2005; Moschos et al., 2008; Choi et al., 2011; Lin et al., 2020; Vargas et al., 2023b). Experimental evidence, including our previous work, has demonstrated that CSI may induce intracranial hypertension. However, the relative contribution of irrigation pressure and flow rate, saline outflow obstruction, surgical duration, dural integrity, and anesthetic management remains incompletely understood.

The present study, together with our previous investigation, demonstrates that CSI can elevate intracranial pressure to critical levels, particularly when saline outflow is compromised, especially at higher pump settings (Amato et al., 2023). These findings provide a plausible physiological explanation for previously reported neurological complications such as headache, neck pain, hypoacusis, and paresthesias (Joh et al., 2009; Flaviano and Bellini, 2018), as well as rare but more severe complications including visual loss and retinal hemorrhage (Gill and Heavner, 2005; Moschos et al., 2008), and epileptic seizures (Choi et al., 2011; Lin et al., 2020). Although derived from a swine model, these findings are consistent with human studies demonstrating a relationship between irrigation and epidural pressure. Joh et al. reported that higher irrigation rates were associated with neck pain and that epidural pressure at symptom onset was significantly higher than the maximal pressure recorded in asymptomatic patients (Joh et al., 2009). Similarly, Choi et al. found an association between neck pain, irrigation rate, procedure duration, and elevated epidural pressure (Choi et al., 2011). Lin et al., evaluating risk factors for seizures during FESS, reported that continuous epidural saline infusion may compress the thecal sac and increase cerebrospinal fluid pressure, particularly during prolonged procedures. The authors identified operative duration and irrigation pressure as relevant modifiable factors and recommended shorter procedures and lower irrigation pressures to reduce complications (Lin et al., 2020). These clinical observations reinforce the need to understand the hydrodynamic and physiological factors governing intracranial pressure during FESS.

### 4.2 Cervical FESS, partial and total outflow obstruction

As cervical FESS becomes increasingly incorporated into clinical practice, irrigation-related ICP changes may be particularly relevant because of the closer anatomical relationship to the intracranial compartment (Komp et al., 2018; Bucknall and Gibson, 2018; Wu et al., 2018; Carr et al., 2020). In the present cervical model, ICP reached ≥20 mmHg immediately after spinal canal opening in three of four pigs, even under the lowest pump configuration (60 mmHg/350 mL/min) and with patent drainage. Consistent with our previous lumbar findings, ICP increased markedly when saline outflow was obstructed. Total occlusion of the drainage channels (TODC), a maneuver that may be temporarily used to improve visualization or control bleeding (Amato, 2023), produced critical ICP elevations even under lower pump configurations in all but the second pig.

Importantly, when both available outflow pathways remained patent and no instrument occupied the working channel (OpDC), increasing pump settings did not result in a proportional increase in ICP. Across configurations A–D, peak ICP values remained within a relatively narrow range in all animals. For example, in pig 3 during mild sedation, peak ICP decreased from 26.2 mmHg in configuration A to 19.7 mmHg in configuration D despite the higher pump settings. These findings suggest that preservation of saline outflow is a key determinant of ICP stability and that pump pressure or flow alone does not predict the intracranial response. This observation has direct technical implications, emphasizing the importance of maintaining drainage patency.

Conversely, drainage obstruction produced marked and sometimes critical ICP elevations. In pig 1, TODC increased peak ICP from 40.4 mmHg in configuration A to 66, 80.1, and 102 mmHg in configurations B, C, and D, respectively. Critical elevations were also observed in pigs 3 and 4, although the magnitude of the response varied among animals. This inter-animal variability suggests that physiological factors, including intracranial compliance and anesthetic conditions, may modify the response to similar hydrodynamic challenges.

Partial outflow obstruction was also clinically relevant. As observed in our previous study, the use of bulky instruments such as Kerrison punches and endoscopic burrs reduced outflow through the working channel and produced substantial ICP elevations (Amato et al., 2023), in some instances approaching those observed during total occlusion (Fig. 4). Unlike complete outflow occlusion, which is relatively uncommon in routine surgery, transient reduction of drainage during instrument use is inherent to many standard maneuvers. These subtle and often unrecognized episodes may therefore represent a clinically relevant mechanism of repeated ICP elevation during endoscopic procedures without the surgeon's awareness.

### 4.3 Anesthesia and physiological modulation of ICP

In our previous porcine experiments, similar intraoperative events produced different ICP responses across animals, raising the possibility that anesthetic management may also influence the intracranial response to irrigation and outflow obstruction. Reports of complications potentially related to intracranial hypertension during FESS have involved local anesthesia, mild sedation, or sevoflurane-based general anesthesia (Flaviano and Bellini, 2018; Gill and Heavner, 2005; Moschos et al., 2008; Choi et al., 2011; Lin et al., 2020). In contrast, propofol- and opioid-based anesthesia are associated with reduced cerebral blood flow (Sahay et al., 2018; Petersen et al., 2002; Borrelli et al., 2016; Yu et al., 2019), and lower ICP compared with volatile anesthetic techniques (Chui et al., 2014), as well as improved analgesia (Peng et al., 2016). Supporting a possible influence of anesthetic state, our previous clinical study using noninvasive ICP monitoring demonstrated reductions in ICP waveform parameters after anesthetic induction and throughout FESS (Ribeiro et al., 2024).

As detailed in the Results, anesthetic depth was associated with ICP behavior and variability (Table 1, Fig. 4, Fig. 5). Deeper anesthesia was associated with lower and less variable ICP responses, particularly during outflow obstruction. This pattern was most evident in pigs 3 and 4, in which different anesthetic depths could be evaluated within the same animal. Pig 2, maintained under stable deeper anesthesia, showed minimal ICP variability throughout the experiment, whereas pig 1, in whom deeper physiological suppression could not be achieved despite high anesthetic doses, exhibited higher and more sustained ICP elevations. In pig 4, deeper anesthesia induced apnea requiring manual ventilation, and increased ventilation was followed by a prompt ICP reduction, indicating that anesthetic depth and respiratory effects cannot be considered fully independent variables. Overall, these findings are consistent with our previous noninvasive ICP study showing reduced ICP after anesthetic induction. They also parallel our clinical experience: after progressively transitioning from predominantly transforaminal procedures under local anesthesia and mild sedation to performing most procedures under propofol-remifentanil TIVA with Bispectral Index monitoring, we observed fewer reports of postoperative headache and neck pain. However, given the small sample and the observational nature of these clinical impressions, these findings should be considered exploratory and warrant further investigation.

### 4.4 Clinical implications

A major concern during FESS is inadvertent dural laceration, as continuous irrigation may directly transmit fluid into the subarachnoid space and influence intracranial pressure (Moschos et al., 2008). Vargas et al. reported three cases of seizures associated with dural tears during FESS, including subarachnoid hemorrhage and pneumocephalus (Vargas et al., 2023a, 2023b). In our previous lumbar model, irrigation could be maintained despite dural tears without sustained or critical ICP elevations when saline outflow remained patent. These observations suggest that the risk associated with dural tears may depend not only on dural integrity itself but also on the prevailing hydrodynamic conditions and drainage patency. Intentional dural laceration was not performed in the present cervical model.

From a practical standpoint, continuous irrigation behaves as an open-flow system only while effective saline outflow pathways remain patent. Any intentional or inadvertent compromise of drainage, whether by finger occlusion, rubber caps, bulky instruments, bone debris, or even partial channel narrowing, can rapidly increase epidural pressure and consequently ICP, even with intact dura. Endoscopes equipped with dedicated drainage channels should therefore be periodically checked to ensure that these pathways are not obstructed by surgical debris and flushed when necessary to restore adequate outflow. Although temporary outflow occlusion is an effective maneuver to control bleeding or facilitate dissection, it should remain brief and be followed by prompt restoration of drainage. Longer procedures, dural tears, and repeated partial obstructions may expose patients to cumulative ICP elevations that occur without visual warning signs. Surgeons should also be aware that different endoscope designs, working-channel diameters, drainage-channel dimensions, and system resistance may substantially alter the relationship between pump settings and actual operative-field pressure. Pump parameters used with one endoscopic system should therefore not be directly extrapolated to systems with different irrigation and outflow characteristics.

Anesthetic management should be considered together with these technical factors. Even though ICP is not routinely monitored during FESS, prolonged drainage obstruction, high pump settings, dural tears, and extended operative times should alert the surgical team to the possibility of intracranial hypertension. Close collaboration with the anesthesia team, use of the lowest effective irrigation settings, and strict attention to drainage patency represent practical measures that may reduce the risk of critical ICP elevations.

### 4.5 Study limitations

The present findings should be interpreted within the context of an experimental porcine model with a limited sample size, and direct extrapolation to human surgery should therefore be approached cautiously. Nevertheless, invasive and continuous ICP monitoring with a gold-standard intraparenchymal catheter would be ethically unjustifiable in patients undergoing routine FESS, making an animal model the most feasible method to directly characterize ICP behavior during epidural irrigation. The anatomical similarity of the spinal canal and epidural space between pigs and humans, together with the reproducibility of ICP responses across our previous and current studies and their consistency with reported clinical complications, supports the physiological relevance of the model. Inter-animal variability and the acute experimental setting may nevertheless have influenced the magnitude of individual ICP responses.

Methodological aspects may also have influenced the magnitude of the recorded responses. Ventilation was manually assisted when required, and continuous capnography was not employed; therefore, transient variations in CO_2_ levels may have contributed to ICP variability. Blood pressure monitoring was noninvasive, limiting the ability to capture rapid hemodynamic changes associated with abrupt ICP elevations. Additionally, it was not feasible to systematically test all anesthetic depth conditions in every animal, and anesthetic adjustments were occasionally required in response to critical ICP elevations or physiological instability. Thus, anesthetic depth, ventilation, and their effects on cerebral physiology could not be fully separated in this model, and the association between deeper anesthesia and attenuated ICP responses should be considered exploratory. Finally, the experiments were performed using a single endoscopic irrigation system, and different endoscope designs and outflow characteristics may produce different hydrodynamic responses. Despite these constraints, the consistent pattern of ICP elevation under defined irrigation and outflow conditions supports the internal validity and physiological coherence of the findings.

## 5 Conclusion

This study demonstrates that continuous saline irrigation during cervical FESS can produce clinically relevant ICP elevations, particularly when saline outflow is compromised. Compared with our previous lumbar model, ICP elevations during cervical FESS occurred earlier and reached higher levels, including under partial or total outflow obstruction. Maintenance of patent drainage limited ICP elevation even at higher pump settings, indicating that outflow conditions are a key determinant of intracranial pressure behavior during irrigation. The findings identify saline outflow compromise as the principal trigger of critical ICP surges, while pump parameters modulate the magnitude of these elevations rather than acting independently.

Deeper anesthesia was associated with lower baseline ICP and attenuation of peak elevations during outflow obstruction, whereas lighter sedation was associated with greater pressure responses, although these exploratory observations require confirmation in larger controlled studies. These findings suggest that the safety of cervical FESS may depend not only on irrigation parameters but also on adequate anesthetic management. From a practical standpoint, maintaining drainage patency, minimizing partial or total outflow obstruction, and using the lowest effective irrigation settings appear to be important measures for reducing the risk of critical ICP elevations during cervical FESS.

## Ethics declaration

This study was conducted in accordance with the ARRIVE (Animal Research: Reporting of In Vivo Experiments) guidelines. This study was approved by the CEUA - Institutional Animal Care and Use Committee. (Approval No. 1058/2022)

## Declaration of generative AI and AI-assisted technologies in the manuscript preparation process

During the preparation of this work, the authors used an AI-assisted language tool to improve clarity and grammar in the English writing. The authors reviewed and edited the content as needed and take full responsibility for the final version of the manuscript.

## Funding

No funding was received for this research.

## Declaration of competing interest

The authors declare the following financial interests/personal relationships which may be considered as potential competing interests: Marcelo Campos Moraes Amato reports equipment, drugs, or supplies was provided by DoppKon GmbH & Co. KG. Marcelo Campos Moraes Amato reports a relationship with joimax GmbH that includes: consulting or advisory, speaking and lecture fees, and travel reimbursement. Ricardo Santos de Oliveira reports article publishing charges was provided by Division of Neurosurgery Ribeirão Preto Medical School University of Sao Paulo. If there are other authors, they declare that they have no known competing financial interests or personal relationships that could have appeared to influence the work reported in this paper.
